# Primary cutaneous nocardiosis: a diagnosis of consideration in a renal transplant recipient

**DOI:** 10.1186/s12907-018-0075-2

**Published:** 2018-10-01

**Authors:** Priyatam Khadka, Dibya Singh Shah

**Affiliations:** 1 Department of Microbiology, Tribhuvan University Teaching Hospital, Kathmandu, Nepal; 2 Medical Microbiology, Tri-Chandra Multiple Campus, Tribhuvan University, Ghantaghar, Kathmandu, Nepal; 3 Department of Nephrology and Renal Transplantation Medicine, Grande International Hospital, Kathmandu, Nepal

**Keywords:** Cutaneous nocardiosis, Cutaneous tuberculosis, Immunocompromised, *Nocardia asteroides*, Renal transplant, Sulfamethoxazole and trimethoprim

## Abstract

**Background:**

The cutaneous nocardiosis remains a diagnostic challenge: similar clinical presentations as of cutaneous diseases with different etiology—and the inherent difficulty in cultivating the pathogen.

**Case presentation:**

Herein, we describe a case of primary cutaneous nocardiosis in a renal transplant recipient; treated with anti-tubercular drugs due to misdiagnosis of cutaneous tuberculosis. On clinical examinations, a few red erythematous papules with erosions and crusting seen, over prior surgery scar of renal transplant. Multiple basophilic colonies surrounded by neutrophilic abscesses and granulation tissue were seen on histopathological examinations. The presumptive identification was done by Ziehl-neelson staining, bacterial culture, biochemical interpretations, and susceptibility pattern of the isolates to antibiotics. Radiographic imaging of brain and lungs were normal; no feature of disseminated nocardiosis seen. After 3 months of an anti-microbial therapy i.e. TMP-SMX(sulfamethoxazole and trimethoprim); the patient underwent progressive changes no relapse noted; transplant function observed in a good state, found asymptomatic with limited side effects on a regular follow up till now.

**Conclusion:**

Cutaneous nocardiosis can occur in the renal-transplant patient. Therefore, a high degree of clinical suspicions, extensive clinical differentiation, early detection of the pathogen, apt selection of the antimicrobial therapy, correct dosing, and treatment duration is crucial for successful outcomes.

## Background

Nocardiosis, the opportunistic infection, most commonly present as pulmonary diseases; however, infrequently may present either as cutaneous or disseminated infections [1]. The rare entity, cutaneous nocardiosis, presents either as a part of disseminated infection or as a primary infection resulting from inoculation [2]. The disease is more frequent in tropical countries, without age or ethnic predilection; although, much common in patients with underlying immunosuppressive therapy for renal transplantation [3]. Moreover, the frequency to acquired nocardiosis varies according to the type of solid organ transplant—highest3.5% in lungs transplant recipient, while lowest (0.004 to 0.7%) in renal transplant; lower the incidence higher the chance of being neglected from diagnosis considerations [4–6].

The cutaneous nocardiosis, in contrast to pulmonary or disseminated forms, in the renal transplant recipient, often leading delay in diagnosis and treatment because of relative paucity of the pathogen *(Nocardia* species), elusive clinical manifestations, difficult microbial entity to grow in-vitro—and appearance on imaging may mimic other entities such as malignancies [3, 7].

We report, herein, a case of primary cutaneous nocardiosis in renal allograft recipient masquerading as cutaneous tuberculosis; was treated successfully with trimethoprim/sulfamethoxazole.

## Case presentation

**Fig. 1:**
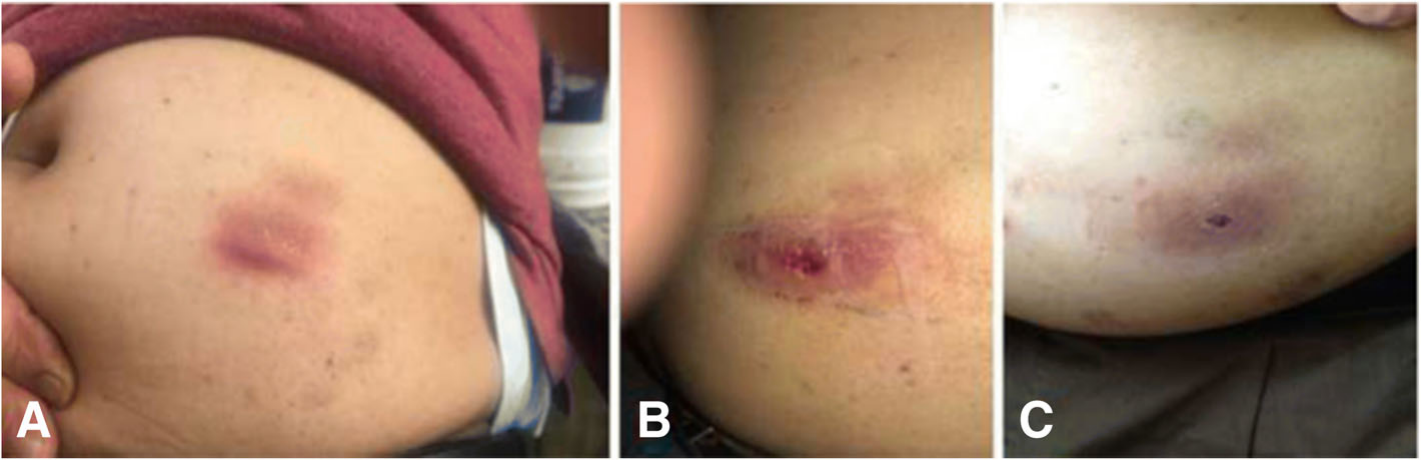
**a** Red erythematous papules (4*3 cm) of cutaneous nocardiosis. **b** Erythematous papules forming the plaque with erosions, pus discharge, and crusting. **c** The erythematous plaque reduced and resolve after successful treatment with TMP-SMX

A 62-year-old Nepali, a professional health worker, presented in Grande International Hospital with red erythematous papules near around the previous site of renal transplantation(Fig. 1a). He had lived with diabetes and hypertension for 27 years and had a history of visiting the south-western and south-eastern region of the USA. The renal transplantation was done 6 months ago (6th October 2017). The previous diagnosis was made as cutaneous tuberculosis; in a local hospital, based on clinical presentations and unresolved lesion with a course of antimicrobial therapy (cloxacillin). Simultaneously, the patient had received a course of anti-tubercular therapy for two months. Paradoxically, the lesions continued to progress with pus discharges. A routine culture revealed no bacterial and fungal growth associated with the infection. On clinical examinations, few red erythematous papules with erosions and crusting seen over the site of prior the scar (Fig. 1b); no other systemic abnormality were found to be associated. Hence, the differential diagnosis of actinomycosis, deep mycosis, and rifampicin resistant cutaneous tuberculosis was made.

### Investigation

On histopathological examination, multiple basophilic colonies surrounded by neutrophilic abscesses and granulation tissue with dense infiltration by acute and chronic inflammatory cells were observed. However, the serological marker: HIV, HBsAg, HCV (ELISA) were negative. The Renal function and Liver function were found normal. The level of tacrolimus was assessed 9.0mcg/dl.

**Fig. 2:**
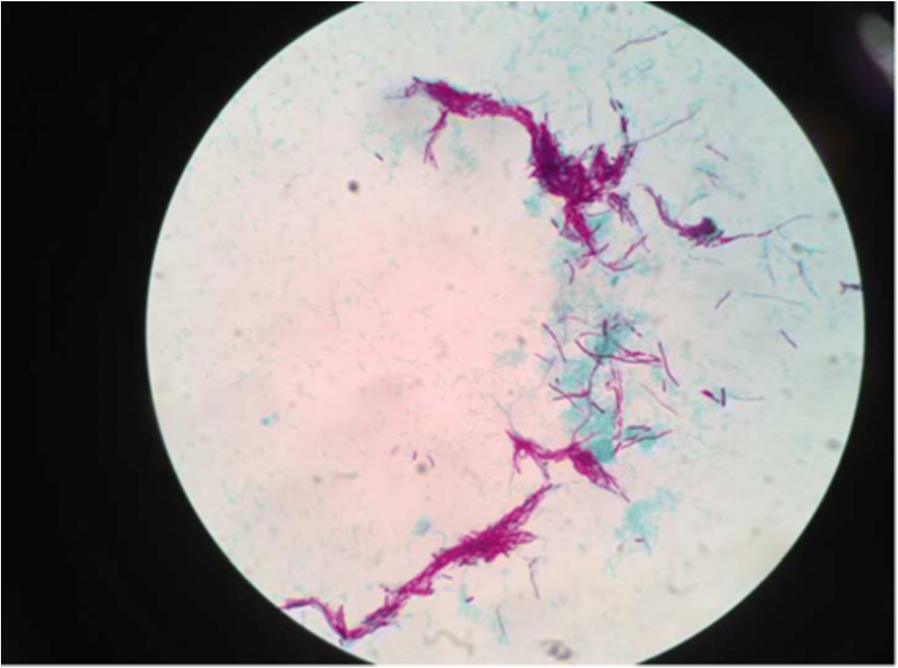
AFB staining: partially acid-fast branching rod suggestive *Nocardia* species on modified. Kinyounstain (1000× original magnification)

**Fig. 3:**
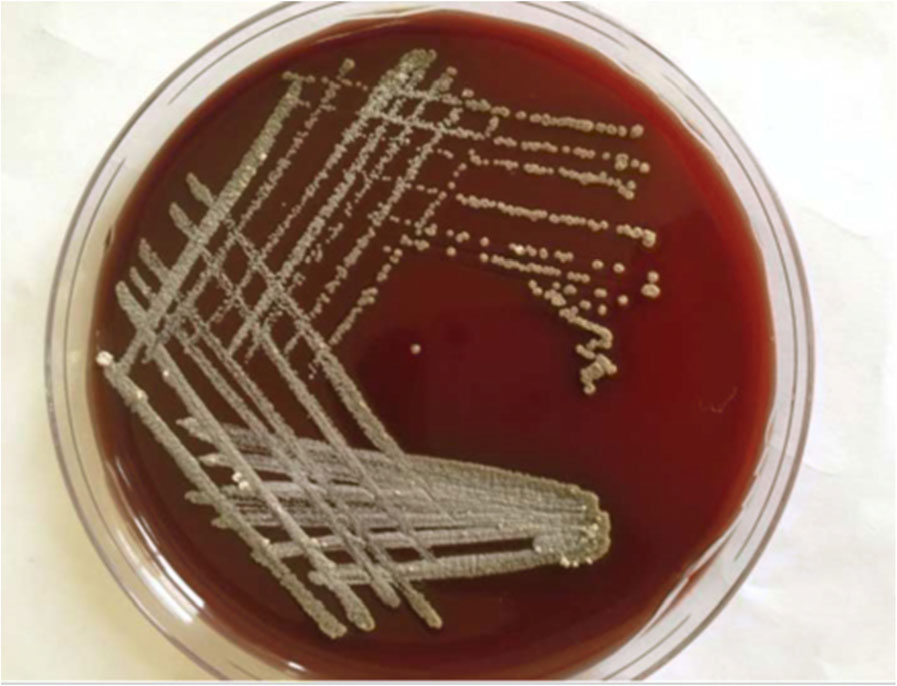
Colonial morphology of *Nocardia* species on blood agar: whitish chalky adherent colonies of *Nocardia* species

**Fig. 4:**
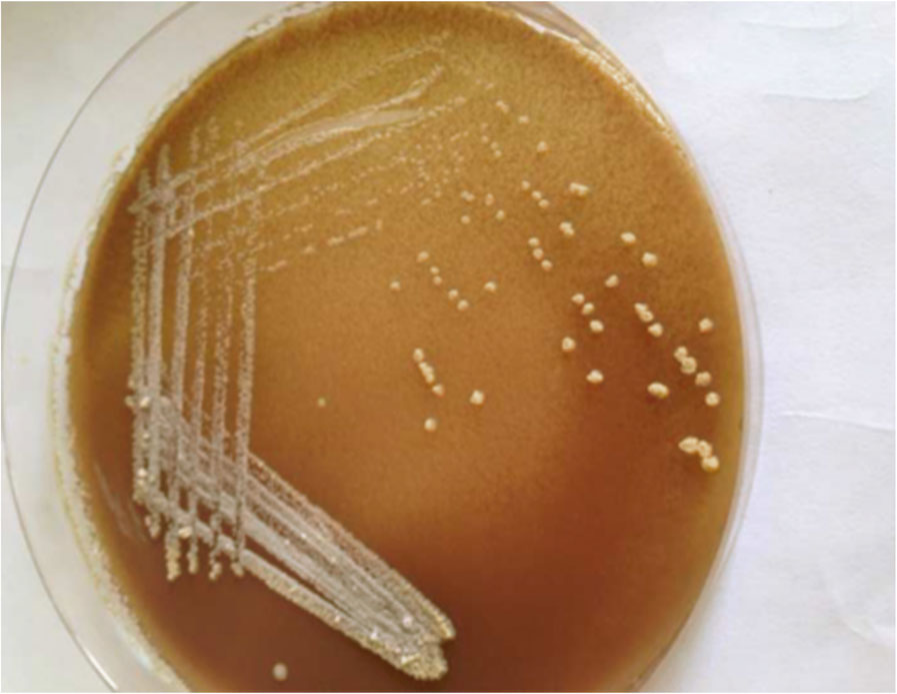
Colonial morphology of *Nocardia* species on chocolate agar: whitish chalky adherent colonies of *Nocardia* species

As of microbiological approaches, for the detection of an etiologies: right angaled branching, filamentous Acid Fast Bacilli (AFB) was observed on Ziehl-neelson staining (Fig. 2); chalky white adherent colonies were seen on aerobic culture after 72 h of incubation, which turns molar tooth appearance on further incubation (Figs. 3 and 4). Further, identification of the isolate, *Nocardia asteroids*, was done with standard microbiological culture methods as recommended by American Society for Microbiology, based on phenotypic characteristics, biochemical interpretations, and varied incubation temperature to grow the pathogen; since molecular analysis and sequencing was not accessible in our laboratory setting. Plus, antibiogram of the isolate was determined by modified Kirby-Bauer disk diffusion method on Blood agar against commercially prepared antibiotic disks (HiMedia Laboratories Pvt. Limited, India) in acquiescence with Clinical Laboratory Standards Institute (CLSI). Additionally, the CT scan of lungs and brain was done to rule out the possible disseminated nocardiosis; however, no abnormalities detected. In view of examination and investigations, a diagnosis of primary cutaneous nocardiosis was made on post-renal transplant patient. Subsequently, anti-tubercular therapy was discontinued, and the patient was treated with trimethoprime and sulfamethoxazole as per the drug susceptibility testing.

### Treatment

The patient was treated with trimethoprime and sulfamethoxazole, BACTRIM-DS (PO × BD), 2 double strength oral tablets each containing 800 mg sulfamethoxazole and 160 mg trimethoprim for 3 months.

### Outcomes and follow-up

He has now completed 2 months of treatment; has undergone progressive changes no relapse noted (Fig. 1c); transplant function observed in a good state. He is under regular follow up since then and we found him asymptomatic; however, with limited side effects due to prolonged antimicrobial therapy.

## Discussion

Cutaneous nocardiosis is a rare infectious disease, presents as primary cutaneous infection or as a part of disseminated pulmonary nocardiosis [2]. The acquisitions of disseminated infection occur via inhalation of the pathogens which in-turn possibly metastasizes haematogenously to distant organs system (lungs, central nervous system, eyes, kidneys, skin, subcutaneous tissue, and bone); nevertheless, inoculation of the pathogen after trauma, surgery, through a vascular catheter or by stings or scratches of animals may develop primary cutaneous nocardiosis [2, 4, 8–10].

Nocardiosis has been observed as an emerging infectious disease, probably due to increasing trend of treating the patient with immunosuppressive therapy and underlying diseases like HIV infection, diabetes, autoimmune disease, COPD, organ transplantation, lymphoreticular neoplasms [7, 10]. The high calcineurin inhibitor trough levels in months before diagnosis, use of tacrolimus and corticosteroid dose at the time of diagnosis, patient age, length of stay in intensive care unit after the solid organ transplant are independently associated with the development of nocardiosis [6, 10]. Relating to our case; post-renal transplantation, lived with diabetes and hypertension, use of tacrolimus and corticosteroid dose at the time of diagnosis, patient age, and length of stay in intensive care unit after solid organ transplant could be the risk factor to acquire the infection.

The clinico-pathological manifestations of cutaneous nocardiosis are diverse. The precise diagnosis is often a challenge, due to its similar clinical presentations with other cutaneous diseases of different etiology and the relative difficulty in isolation of the pathogens in lesions [2]. In present case, the infections masqueraded as cutaneous tuberculosis preceding characteristic erythematous papules which eventually coalesced into plaques. Nevertheless, the clinical form masquerading: atypical zygomycosis, superficial celluitis or localized abscesses, lymphoadenities or lymphocutaneous infection, and even generalized limbs swelling; has also been reported in renal transplant recipient with presumed cutaneous nocardiosis [11–13]. The culture revealed no other bacterial and fungal growth in routine culture procedures (incubated for 24-48 h); however, the patient was treated empirically with anti-tubercular therapy for months, based on clinical presentations. The presumptive diagnosis could have made earlier with Ziehl-neelson staining and then with precise culture procedures implicated to *Nocardia* species, nevertheless. Therefore, when a persistent no growth on a clinical sample and impassive anti-tubercular regimen attributed; a differential diagnosis of cutaneous nocardiosis should be made.

The identification of *Nocardia* species in the clinical laboratory is challenging: may appear gram-negative and acid-fast-stain negative, require longer incubations period for growth [14]. In these perspectives, the clinicians must resort to every possible test, so that supporting positive rudiments would be an ancillary in the early and precise diagnosis of cutaneous nocardiosis. The presumptive identification of our case was done by AFB staining, bacterial culture with an extended incubation period, biochemical interpretations, and susceptibility pattern of the isolates to antibiotics; sequencing of 16SrRNA was not available in our setting, however.

Patient with primary cutaneous nocardiosis responds well to medical therapy. However, early detection of the pathogen, apt selection of the antimicrobial therapy, correct dosing and treatment duration is crucial in a successful management and treatment in cutaneous nocardiosis; limiting the tendency of late relapse [15, 16]. Trimethoprim-sulfamethoxazole was chosen as a suitable antimicrobial therapy in our case, because of its good bioavailability in all tissue including the worst irrigated to bones, plus of its greater efficiency against most of the *Nocardia* species [4, 10]. In addition, the patient receiving TMP-SMX confer lower relapse rate of about 13.6% (compared to 32% mortality rate and 16% relapse rate in patients who did not receive TMP-SMX) [3, 17, 18]. The supplementary or alternate parenteral antimicrobial therapies—carbapenems (imipenem or meropenem, but not ertapenem), third-generation cephalosporins (cefotaxime or ceftriaxone), and amikacin, alone or in combination—although, recommended by some authors were thought unnecessary, because of possible toxicity relating to these drugs: cephalosporins and aminoglycosides—alone or in combinations—(nephrotoxic), carbapenem (myelosupression, optic nerve damage and lactic acidosis), linezolid (hematologic toxicity, lactic acidosis, optic neuritis or peripheral neuropathy) [3, 4, 18–22]. Scrutinizing these prospective, TMP-SMX has been considered, the appropriate treatment of choice. The clinical response to therapy of BACTRIM-DS (PO × BD), 2 double strength tablets each containing 800 mg sulfamethoxazole and 160 mg trimethoprim was found evident within five days of medication in our case, even though the medications were continued for 3 months.

Moreover, the relative rarity of the cases, non-specific clinico-pathological presentations, diagnostic intricacies, and with lack of systematic reporting; the exact burden of nocardiosis in low-income countries like Nepal is not clear but speculated to be small.

## Conclusion

The challenges in establishing the precise diagnosis of cutaneous nocardiosis often encountered, in clinical practice, due to the relative paucity of the pathogen in lesions, multifaceted clinico-pathological features as of cutaneous diseases with different etiology, and inherent difficulty in-vitro cultivating. Henceforth, particularly in the renal transplant recipient, a high degree of clinical suspicions, early detection of the pathogen, apt selection of the antimicrobial therapy, correct dosing and treatment duration is crucial for successful outcomes.

## Abbreviation

AFB: Acid Fast Bacilli
ATT: Anti-Tubercular Treatment
CLSI: Clinical and Laboratory Standard Institute
COPD: Chronic Obstructive Pulmonary Disease
CT: Computerized Tomography
HBsAg: Hepatitis B surface antigen
HCV: Hepatitis C Virus
HIV: Human Immune deficiency Virus
LFTs: Liver Function test
RFT: Renal Function test
TMP-SMX: Trimethoprim-sulfamethoxazole
